# Prevalence of osteochondral lesions in the fetlock and hock joints of Standardbred horses that survived bacterial infection before 6 months of age

**DOI:** 10.1186/s12917-018-1726-3

**Published:** 2018-12-10

**Authors:** Eli H. S. Hendrickson, Sigrid Lykkjen, Nils I. Dolvik, Kristin Olstad

**Affiliations:** Equine Section, Department of Companion Animal Clinical Sciences, Faculty of Veterinary Medicine, Norwegian University of Life Sciences, Ullevålsveien 72, 0454 Oslo, Norway

**Keywords:** Bacteria, Epiphyseal growth cartilage, Horse, Ischaemic chondronecrosis, Osteochondral fragment, Osteochondrosis, Osteochondrosis dissecans, Prevalence, Sepsis

## Abstract

**Background:**

Young Standardbred horses frequently develop fragments in joints. Some fragments represent osteochondrosis; others are considered developmental, but it is uncertain whether they result from preceding osteochondrosis. Osteochondrosis occurs as a consequence of failure of the cartilage canal blood supply and ischaemic chondronecrosis. In heritably predisposed foals, failure was associated with incorporation of vessels into bone. However, bacterial vascular failure was also recently documented in foals suffering spontaneous infections, proving that bacteria can cause osteochondral lesions in foals up to 150 days old. The aim was to determine prevalence of fetlock and hock lesions at screening age in Standardbred horses that survived infections before 6 months of age, and compare this to prevalence reported in the literature.

**Methods:**

The material consisted of 28 Standardbred horses; 17 males and 11 females that presented and were diagnosed clinically with bacterial infections from 1 to 150 days of age (average: 41.3 days). A screening set of 8 radiographic projections was available from all 28 horses at 7–85 months of age (average: 23.6 months). Lesion prevalence was compared to three previously reported Standardbred cohorts.

**Results:**

Osteochondral lesions were detected in one or more joints of 19/28 horses (67.9%); in the fetlock joint of 14/28 horses (50%) and the hock joint of 11/28 horses (39.3%). These prevalences were ≥ 2 x higher than the corresponding prevalences in the comparison cohorts, and statistically significantly so in 5:6 comparisons (*p*-values from < 0.00001 to 0.01). In the sepsis cohort, there were an average of 2.3 affected joints and 2.5 lesions per affected horse, whereas there in the one comparable literature cohort were an average of 1.5 affected joints and 1.7 lesions per affected horse.

**Conclusions:**

Standardbred horses that survived bacterial infections before 6 months of age had more osteochondral lesions than literature comparison cohorts at screening age. The implication was that some of the lesions in this group were caused by bacteria. It may become necessary to develop methods for differentiating between acquired, septic and aseptic, heritably predisposed lesions.

**Electronic supplementary material:**

The online version of this article (10.1186/s12917-018-1726-3) contains supplementary material, which is available to authorized users.

## Background

The pathogenesis of articular osteochondrosis in horses has been documented at three levels of evidence: changes are present at predilection sites prior to clinical lesions [1, 2], identical changes occur in more than one species [2, 3], and lesions can be experimentally reproduced [4, 5].

The pathogenesis starts with focal failure of the cartilage canal end arterial blood supply to epiphyseal growth cartilage [6]. The morphology of spontaneous lesions in both heritably predisposed foals [2] and pigs [3] indicates that failure occurs where the mid-portion of blood vessels is incorporated into the advancing ossification front. This results in ischaemic necrosis of chondrocytes at mid-depth of the growth cartilage [2, 3]. With time, the area of ischaemic chondronecrosis, or infarct, is surrounded by the ossification front, where it causes the focal delay in endochondral ossification that is characteristic of osteochondrosis [2, 3]. Lesions can resolve [7–9], or pathologic fracture can occur through the infarct, leading to fragments in joints known as osteochondrosis dissecans (OCD) [5, 10]. Fragments at predilection sites in the hock and stifle are referred to as OCD because it has been confirmed that they are the result of preceding osteochondrosis [1, 2]. In the fetlock, there is consensus that fragments at the sagittal ridge of the third metacarpal/metatarsal bone represent OCD [11, 12]. In the three other commonly affected sites, dorso-proximal (D) on the first phalanx (P1; DP1 fragments), axially on the palmaro−/plantaro-proximal border of P1 (synonyms: type I [13], Birkeland fracture [14], palmaro−/plantaro-proximal osteochondral fragment; POF) and at the abaxial, palmaro−/plantaro-proximal eminence of P1 (synonyms: type II [13], wing fracture [15], ununited palmaro−/plantaro-proximal eminence; UPE) lesions are referred to as osteochondral fragments because although it is agreed they are developmental, it is uncertain whether they are the result of preceding osteochondrosis [8, 16]. Heritability has been estimated for all hock and fetlock fragments [17–19] except UPEs, omitted due to low prevalence.

Since the pathogenesis was experimentally reproduced in 2013 [5], it is necessary to discover the exact cause of vascular failure in heritably predisposed osteochondrosis. Comparative literature search revealed an already-documented cause in pigs [20, 21] and chickens [22–24]. Bacteria injected into the circulation or joints of skeletally immature individuals colonise the cartilage canal blood supply, where they cause obstruction and vascular failure [20–23]. Bacterial vascular failure was also recently documented in foals suffering spontaneous infections [25]. Septic failed cartilage canals were differentiated from aseptic failed canals based on presence of bacteria and perivascular neutrophils [25]. In the studied foals, septic vascular failure was associated with cartilage infarction, delayed ossification and pathologic fracture [25], i.e. the same pathogenesis as described for aseptic, heritably predisposed OCD.

The study of spontaneous infections documents that bacteria can cause osteochondral lesions in foals before 150 days/4.9 months of age [25]. This immediately raises the question: do horses that survived bacterial infections as foals have increased prevalence of osteochondral lesions at screening age for heritably predisposed disease? If so, the spontaneous infections study supports that some of those lesions were caused by septic, rather than heritably disposed vascular failure [25]. This leads to false positive diagnosis of an individual as a carrier of genetic disease, when the true cause of lesions is acquired bacterial infection. False diagnoses are important because they can cause selection strategies to fail in reducing disease prevalence. False negative diagnosis due to lesions resolving before screening is at least part of the explanation for why prevalence of hock lesions increased from 14% to > 31% in Dutch Warmbloods between 1987 and 2009 when osteochondrosis-positive stallions were excluded from breeding [26, 27]. False diagnosis of an individual as a disease-gene carrier would not increase prevalence of heritable disease, but it would result in unwarranted exclusion of potentially valuable individuals and reduced gene pool, including in small breeds with already-high inbreeding coefficients like the Norwegian-Swedish Coldblooded trotter [28].

The aim of the current study was to determine the screening age prevalence of fetlock and hock lesions in Standardbred horses that survived infections before 6 months of age, and compare this to prevalence reported in the literature.

## Methods

### Sepsis cohort

The study cohort was recruited from the equine teaching hospital of the Norwegian University of Life Sciences. All Standardbred foals of both sexes that were ≤ 6 months old at the time of presentation to the hospital during 2006–2012 were identified. Foals without infections were excluded. Criteria for identifying foals with bacterial infections were clinical diagnoses of sepsis, omphalitis, septic arthritis, pneumonia and diarrhoea/enteritis, supported by bacterial culture results in most cases and sepsis scoring according to Brewer [29] in 10/28 cases. Foals that did not survive to be discharged from hospital, or were otherwise unavailable for radiographic screening at ≥7 months old [8] were excluded. The remaining foals (*n* = 28) were included in the study sepsis cohort, and all available clinical records and radiographs were collected for each foal.

### Radiographic screening

A standard set of 8 radiographic projections was already available, or was obtained from the sepsis cohort at age ≥ 7 months old [8]. The set comprised: 2 latero-medial projections of the front fetlocks, 4 dorsal-35°-proximal-45°-lateral and –medial oblique projections of the hind fetlocks, and 2 dorsal-45°-medial oblique projections of the hocks. In a previous study, prevalence of medial malleolar lesions was ≤2.5% [30], thus acquisition of a separate dorsal-30°-lateral oblique projection to detect lesions in this site was not considered justified. Additional radiographic projections including serial examinations were available from 7/28 horses.

### Radiological evaluation

Radiographs were evaluated by authors EH and NID. Six predilection sites were evaluated in each fetlock, comprising the sagittal ridge of the third metacarpal/metatarsal bone, dorso-proximal border of P1, and the axial palmaro−/plantaro-proximal border and abaxial palmaro−/plantaro-proximal eminence on both the medial and lateral side of P1. In front fetlocks, lesions were occasionally visible along the palmaro-proximal border of P1 in lateral views and additional oblique projections were obtained to determine if a lesion was medial or lateral. In hind fetlocks, it was possible to detect dorsal lesions superimposed on the third metatarsal bone by adjusting window levels. Three predilection sites were evaluated in the hock, comprising the cranial distal intermediate ridge of the tibia, and the lateral and medial trochlear ridges of the talus.

**Fig. 1:**
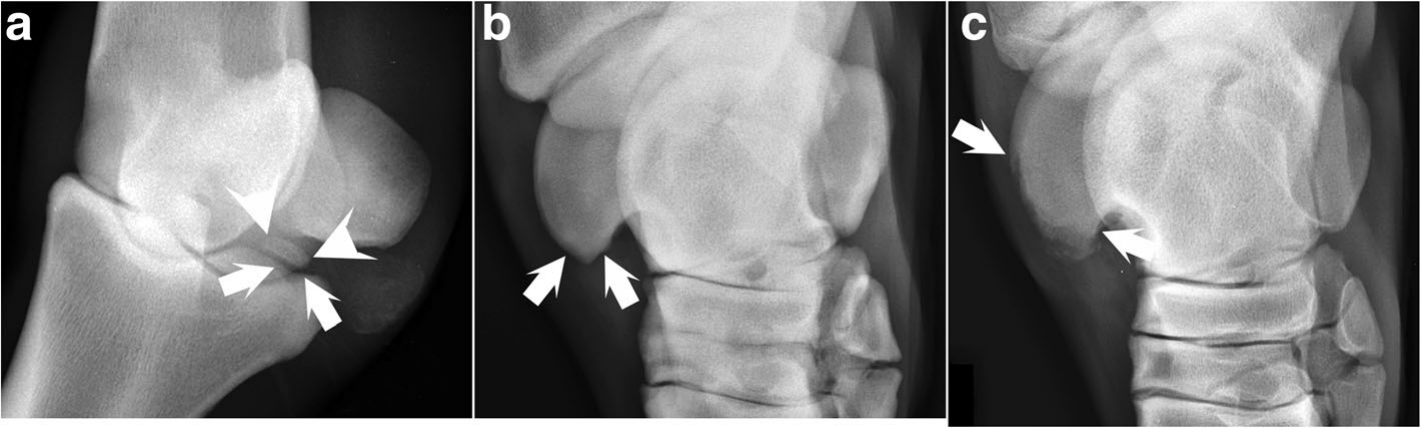
Categories of change registered. **a** Dorsal-35°-proximal-45°lateral projection of the left hind fetlock of horse 15. There is a focal, uniformly radiolucent defect (between arrows) in the lateral half of the plantaro-proximal contour of the first phalanx, referred to as a fragment bed. There is also a mineralised body (between arrowheads), referred to as a fragment. **b** Dorsal-45°-medial oblique screening projection of the left hock of a female Warmblood horse at 46 months old, diagnosed with sepsis at 6 days old; there is a pointy, bone radiopaque protrusion (between arrows) at the distal end of the lateral trochlear ridge of the talus, referred to as a spur. **c** At 4 months and 10 days old, the projection demonstrates focal, radiolucent defects (between arrows; osteochondrosis) in the contour at the same site as the spur in **b**

Three categories of radiographic change were evaluated (Fig. 1). Two categories were based on the existing literature: focal, uniformly radiolucent defects in the bone contour, referred to as fragment beds (Fig. 1a), and mineralised bodies in or adjacent to the bone contour, referred to as osteochondral fragments (Fig. 1a). When a fragment bed was present alone, it was counted as 1 lesion. When an osteochondral fragment was present, it was counted as 1 lesion and the presence of an associated fragment bed was noted, but not counted as a separate lesion. If there were > 1 fragment bed or > 1 fragment present within a single affected site, these were counted as 1 lesion but the total number of beds and fragments was noted. The third category of change was discovered during case recruitment and previously undescribed. It consisted of a rounded or pointy, drop- or spur-shaped bone radiopaque protrusion at the distal end of the lateral trochlear ridge of the talus, referred to as a spur (Fig. 1b). Additionally available serial radiographs confirmed that spurs were located at the site of previous osteochondrosis lesions (Fig. 1c), and spurs were therefore counted as lesions.

### Comparison to literature

The literature was searched for studies reporting prevalence of hock and fetlock osteochondral lesions in young Standardbreds (Additional file 1: Table S1). Studies with < 350 horses and markedly different radiographic projections and scoring were excluded (Additional file 1: Table S1). The search returned three comparison studies: Grøndahl & Dolvik, 1993 [17], Philipsson et al., 1993 [31] and Lykkjen et al., 2012 [30], referred to as the AMG, JP and SL cohorts, respectively. The authors had access to the raw data of the SL cohort [30]. Prevalence, number of affected joints and number of lesions were compared using the chi-square test and a significance level of *p* < 0.05.

## Results

### Sepsis cohort

The sepsis cohort of 28 horses was assigned ascending numbers according to age at presentation, ranging from 1 to 150 days (average: 41.3 days, median: 20 days, Table 1). The cohort included 17 colt and 11 filly foals. Clinical diagnoses, bacterial culture results and sepsis scores are presented in Table 1. Age at radiographic screening ranged from 7 to 85 months (average: 23.6 months, median: 15 months, Table 1).

**Table 1:** Sepsis cohort horses

Horse number	Age at admission	Sex	Primary diagnosis	Secondary diagnoses	Bacterial culture results	Sepsis score	Age at radiographic screening
1	1 day	Male	Sepsis	Septic arthritis L & R TCJ	Negative	14	21 months
2	1 day	Male	Sepsis	ALD LH, tendon laxity all 4 limbs	–	11	38 months
3	1 day	Female	Sepsis	ALD RH	*Escherichia coli*	11	10 months
4	1 day	Female	Sepsis	–	*Pseudomonas sp.*	–	48 months
5^a^	1 day	Female	Sepsis	–	*Enterococcus faecalis, Actinobacillus equuli*	9	49 months
6	2 days	Male	Sepsis	Pneumonia	*Escherichia coli*	14	24 months
7	3 days	Female	Sepsis	ALD, tendon laxity all 4 limbs	–	11	28 months
8	7 days	Male	Septic arthritis R FPJ	–	–	7	12 months
9^a^	11 days	Male	Sepsis	–	Gram negative rods	4	13 months
10^a^	11 days	Male	Omphalitis	–	*Streptococcus zooepidemicus*	–	12 months
11^a^	12 days	Male	Sepsis	–	–	13	12 months
12	13 days	Male	Septic arthritis L & R FPJ, L & R TCJ	–	Mixed	6	16 months
13	14 days	Female	Pneumonia	–	*Bordetella bronchiseptica, Pasteurella sp.*	–	48 months
14	14 days	Male	Pneumonia	Omphalitis	–	–	12 months
15	26 days	Male	Pneumonia	–	*Bordetella bronchiseptica*	–	41 months
16	32 days	Female	Pneumonia	–	*Bordetella bronchiseptica, Streptococcus zooepidemicus, Pasteurella sp.*	–	15 months
17	35 days	Female	Pneumonia	–	*Rhodococcus equi*	–	15 months
18	45 days	Male	Pneumonia	–	*Rhodococcus equi*	–	11 months
19^a^	60 days	Male	Pneumonia	Uveitis	*Rhodococcus equi*	–	85 months
20^a^	60 days	Female	Pneumonia	Megaoesophagus	Mixed	–	36 months
21^a^	60 days	Male	Pneumonia	–	*Streptococcus zooepidemicus, Actinobacillus sp.*	–	11 months
22^a^	63 days	Female	Pneumonia	–	*Rhodococcus equi*	–	14 months
23	70 days	Female	Diarrhoea/enteritis	–	*Salmonella typhimurium*	–	12 months
24	90 days	Male	Pneumonia	–	*Actinobacillus sp., Streptococcus zooepidemicus*	–	9 months
25	97 days	Male	Pneumonia	–	*Acinetobacter*	–	9 months
26^a^	127 days	Female	Pneumonia	–	*Streptococcus zooepidemicus*	–	36 months
27	150 days	Male	Pneumonia	–	*Streptococcus zooepidemicus*	–	16 months
28	150 days	Male	Pneumonia	L TCJ effusion	*Actinobacillys equuli, Klebsiella pneumoniae*	–	7 months
Range	1–150 days						7–85 months
Average	41.3 days						23.6 months
Median	20 days						15 months

^a^Lesion negative

### Lesion prevalence at screening age

Nine of the 28 sepsis cohort horses were radiographically negative (Table 1). The age at admission, septic arthritis status, location and character of all lesions in the remaining lesion-positive horses are summarised in Table 2. As 19/28 sepsis cohort horses were positive for lesions in ≥1 joint, the combined hock and fetlock lesion prevalence was 67.9% (Table 3). Clinical diagnoses, sepsis scores and bacterial culture results were similar between horses with and without lesions (Table 1). The corresponding prevalences in the AMG, JP and SL cohorts were 42.2, 35.9 and 50.7%, respectively (Table 3). The sepsis cohort prevalence was statistically significantly higher than the AMG and JP cohort prevalences at *p* = 0.007 and *p* = 0.0005, respectively. The *p*-value for the comparison to the SL cohort prevalence was 0.058, i.e. not statistically significant.

**Table 2:** Sepsis cohort lesions

Horse number	Age at admission	Septic arthritis	Axial dorso-proximal first phalanx (POF)^a^	Sagittal ridge third metacarpal/metatarsal bone	Dorso-proximal first phalanx	Cranial distal intermediate ridge of tibia	Lateral trochlear ridge of talus	Medial trochlear ridge of talus
1	1 day	L^b^ & R^c^ TCJ^d^	–	–	LF^e^ OCF^f^, RF^g^ OCF	–	–	LH^h^ FB^i^, RH^j^ FB
2	1 day		LH lat^k^ OCF	RF FB	–	–	–	–
3	1 day		RH med^l^ OCF	–	–	–	–	–
4	1 day		–	–	LF OCF	–	LH spur	–
6	2 days		RH lat OCF	–	–	–	–	–
7	3 days		LH med OCF, LH lat OCF, RH med OCF***	LF OCF, RF FB	–	–	–	–
8	7 days	R FPJ^m^	LH lat FB	–	–	–	–	–
12	13 days	L & R FPJ, L & R TCJ	LH lat OCF**, RH med OCF, RH lat OCF^##^	–	LF OCF^##^	LH FB, RH OCF	RH OCF	–
13	14 days		LF med OCF	–	–	–	LH spur, RH spur	–
14	14 days		LH med OCF	–	–	–	–	–
15	26 days		LH med OCF, LH lat OCF	–	–	–	–	–
16	32 days		RH med OCF	–	–	LH OCF, RH OCF	–	–
17	35 days		–	–	–	LH OCF	–	–
18	45 days		–	–	–	LH OCF, RH OCF	–	–
23	70 days		LH med OCF, RH med FB	–	–	–	RH spur	–
24	90 days		RH med FB	LF OCF, RF FB	–	–	–	–
25	97 days		–	–	–	LH OCF, RH OCF	–	–
27	150 days		–	–	–	LH OCF, RH OCF	–	–
28	150 days		–	–	–	LH med malleolus OCF ^###^	RH OCF	–

^a^POF: Palmaro−/plantaro-proximal osteochondral fragment. ^b^L: Left. ^c^R: Right. ^d^TCJ: Tarso-crural joint. ^e^LF: Left forelimb. ^f^OCF: Osteochondral fragment. ^g^RF: Right forelimb. ^h^LH: Left hindlimb. ^i^FB: Fragment bed. ^j^RH: Right hindlimb. ^k^Lat: Lateral. ^l^Med: Medial. ^m^FPJ: Femoro-patellar joint. *Number of fragment beds if multiple. ^#^Number of osteochondral fragments if multiple

**Table 3:** Comparison to existing literature

Cohort	Horse prevalence	Fetlock prevalence	Hock prevalence	Bilateral lesions	Location of POF^a^
Sepsis cohort	19/28 horses (67.9%)*	14/28 horses (50%)*	11/28 horses (39.3%)*	Hock lesions: 72.7% Fetlock lesions: 42.8% DP1^b^: 33.3% bilateral POF: 25% bilateral	94% hind limb: 6% fore limb 61% medial: 39% lateral
AMG cohort [17]	318/753 horses (42.2%)*	89/753 horses (11.8%)*	108/753 horses (14.3%)*	Hock OCD: 45.4% bilateral DP1: 12.4% bilateral	94% hind limb: 6% fore limb 76.3% medial: 23.7% lateral
JP cohort [31]	285/793 horses (35.9%)*	178/793 horses (22.4%)*	91/793 horses (11.5%)*	Hock OC: 45% bilateral	95.6% hind limb: 4.4% fore limb 75.5% medial: 24.5% lateral
SL cohort [30]	179/363 horses (50.7%)	84/363 horses (23.1%)*	70/363 horses (19.3%)*	DIRT OCD: 37.1% bilateral POF: 17.9% bilateral	–

^a^POF: Palmaro−/plantaro-proximal osteochondral fragment. *Statistically significantly higher in the sepsis cohort than the comparison cohorts at at p values from < 0.0001 to 0.01. ^b^DP1: Lesions at the dorso-proximal border of the first phalanx

Fourteen of the 28 sepsis cohort horses had a lesion in ≥1 fetlock joint, resulting in a 50% prevalence of fetlock lesions (Table 3). Eleven of the 28 horses had a lesion in ≥1 hock joint, resulting in a 39.3% prevalence of hock lesions (Table 3). The sepsis cohort prevalence was ≥2x any comparison cohort hock or fetlock prevalence, and this was statistically significant in all cases (*p* values from < 0.00001 to 0.01).

It was noted that among the 7 horses that were ≥ 35 days old at presentation, 5 had hock lesions only, 1 had fetlock lesions only and 1 had both hock and fetlock lesions (Table 2). It was also noted that horses 1, 8 and 12 had lesions in multiple joints in addition to ones that had been diagnosed with septic arthritis (Table 2).

The AMG [17], JP [31] and SL [30] cohort descriptions contain slightly different data, thus not all variables/parameters can be compared between all cohorts:

### Symmetry

In the sepsis cohort, 8/11 horses (72.7%) were affected in both hocks (Table 3). This was higher than the reported symmetry of hock osteochondrosis/OCD in the AMG, JP and SL cohorts of 45.4, 45 and 37.1%, respectively (Table 3). Also in the sepsis cohort, 6/14 horses (42.8%) were affected in a pair of fetlocks (Table 3), but symmetry was only reported on a per-lesion basis in the comparison cohorts. Thus, 12.4% of DP1 lesions were bilateral in the AMG cohort and 17.9% of POFs were bilateral in the SL cohort, compared to 33.3 and 25% for very low numbers of the same lesions in the sepsis cohort. Significance was not tested due to the low numbers of lesions in the sepsis cohort.

### Location of POFs

In the sepsis cohort, POFs were 94% in the hind limbs and 61% medial (Table 3). This compares to 94% hind limb and 76.3% medial POFs in the AMG cohort and 95.6% hind limb and 75.5% medial POFs in the JP cohort (Table 3). Thus, all studies agree that POFs are more common medially in the hind limbs, but the proportion of lateral POFs was higher, but not significantly so in the sepsis cohort than the AMG and JP cohorts.

There were minor differences in the relative prevalence of the different fetlock lesions, but these were not compared because of the low number of fetlock lesions in the sepsis cohort, and because they had been registered slightly differently in the comparison cohorts (Additional file 1: Table S1).

The different mechanisms behind aseptic and septic vascular failure raised the question of whether there were more affected joints and more lesions per affected horse in the sepsis cohort. From the AMG cohort, it was possible to extract that there were 157 affected hock joints in 108 horses, i.e. 1.45 affected hock joints per positive horse, but it was not possible to tell which of these horses were also affected in the fetlock [17]. The same was true of the JP [31] and SL [30] cohorts, thus new information was extracted from the SL cohort to make the comparison:

### Number of affected joints per positive horse

In the sepsis cohort 19 positive horses, there were 43 affected joints and an average of 2.3 affected joints per horse (median: 2; 95% confidence interval [CI] 1.72 ≤ x ≤ 2.8), whereas in the SL cohort, there were 268 affected joints in the 179 positive horses, thus 1.5 affected joints per horse (median: 1; 95% CI 1.46 ≤ x ≤ 2.33, Table 4). The distribution of the number of affected joints in the positive sepsis and SL horses is shown in Table 4. The major 73.7% of the sepsis cohort were affected in > 1 joint, whereas the major 60.3% of the SL cohort were affected in 1 joint and this difference was statistically significant at *p* = 0.02.

**Table 4:** Number of affected joints and lesions per positive horse

Group	Total joints	Joints per horse	Horses affected in 1 joint		Horses affected in 2 joints^a^	Horses affected in 3 joints	Horses affected in 4 joints	Horses affected in 5 joints	
Sepsis cohort (*n* = 19)	43	2.3	6 (31.6%)		6 (31.6%)	4 (21%)	2 (10.5%)	1 (5.3%)	
SL cohort (*n* = 179) [30]	268	1.5	108 (60.3%)		58 (32.4%)	8 (4.5%)	5 (2.8%)	–	
Group	Total lesions	Lesions per horse	Horses with 1 lesion	Horses with 2 lesions^b^	Horses with 3 lesions	Horses with 4 lesions	Horses with 5 lesions	Horses with 6 lesions	Horses with 7 lesions
Sepsis cohort (*n* = 19)	47	2.5	5 (26.3%)	7 (36.8%)	4 (21%)	1 (5.3%)	1 (5.3%)	–	1 (5.3%)
SL cohort (*n* = 179) [30]	302	1.7	98 (54.7%)	56 (31.3%)	13 (7.3%)	8 (4.5%)	3 (1.7%)	1 (0.6%)	–

^a^The proportion of horses affected in ≤2 joints was statistically significantly higher in the sepsis cohort than the SL cohort at *p* = 0.02. ^b^The proportion of horses with ≤2 or more lesions was statistically significantly higher in the sepsis cohort than the SL cohort at *p* = 0.02

### Number of lesions per positive horse

There were a total of 47 lesions in the sepsis cohort 19 positive horses and an average of 2.5 lesions per horse (median: 2; 95% CI 1.78 ≤ x ≤ 3.16) compared to 302 lesions in 179 horses and 1.7 lesions per horse in the SL cohort (median: 1; 95% CI 1.54 ≤ x ≤ 1.83; Table 4). The distribution is shown in Table 4, where it was noted that 1 horse in the SL cohort was affected in 6 different joints. The major 73.7% of the sepsis cohort had > 1 lesion, whereas the major 54.7% of the SL cohort had 1 lesion and this difference was statistically significant at *p* = 0.02.

### Character of lesions

**Fig. 2:**
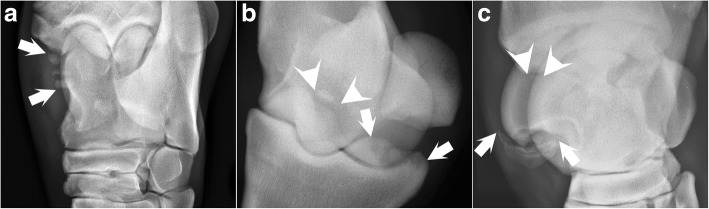
Complex and large lesions in the sepsis cohort. **a** Dorsal-45°-lateral oblique projection of the left hind hock of horse 28. The dorsal-45°-medial oblique projection revealed multiple fragments (between arrows) superimposed on the talus, and this additional projection revealed that the fragments originated from the medial malleolus. **b** Dorsal-35°-proximal-45°-lateral oblique projection of the right hind fetlock of horse 8. Axially, there are two mineralised bodies and three radiolucent defects (between arrows) in lateral half of the plantaro-proximal contour of the proximal phalanx. A third mineralised body (between arrrowheads) is also visible through the third metatarsal bone, located at the medial half of the plantaro-proximal border of the proximal phalanx. **c** Dorsal-45°-medial oblique projection of the right hind hock of horse 8. There is a large mineralised body (between arrows) at the distal end of the lateral trochlear ridge of the talus. There is also a small mineralised body with an associated radiolucent defect (between arrowheads) at the cranial distal intermediate ridge of the tibia

The 47 lesions of the sepsis cohort were distributed as 34 osteochondral fragments with or without fragment beds, 9 lesions with fragment beds only and 4 spurs (Table 2). Horse 28 had multiple fragments superimposed on the talus in the dorsal-45°-medial oblique projection; additional projections revealed that the fragments originated from the medial malleolus (Fig. 2a). This was the only hock lesion with multiple fragments (Table 2). Other than this, horse 7 had 3 fragment beds and 1 fragment medially in the right hind fetlock (Fig. 2b). Horse 12 had 2 fragment beds and 1 fragment laterally in the left hind fetlock, a large fragment bed and 2 fragments laterally in the right hind fetlock, and 2 fragments dorso-proximally at P1 in the left fore fetlock (Table 2). The left hind cranial distal intermediate ridge of the tibia and right hind lateral trochlear ridge of the talus lesions in horse 12 were very large (Fig. 2c), and the right hind lateral fetlock lesion in horse 6 was also large.

## Discussion

The main finding was that the prevalence of osteochondral lesions was higher in the sepsis cohort than in the AMG [17], JP [31] and SL [30] comparison cohorts.

The current study should be considered a preliminary investigation of the effect, if any, of sepsis on lesion prevalence at screening age, in which case validation in larger populations could be warranted. Recruiting the optimal control group would be a challenge. Medicine use including antibiotics is recorded in horse passports, but foals do not acquire passports until they are 3 months old, i.e. too late for validation purposes. Recruitment of horses that did not suffer bacterial infections as foals would therefore only be as reliable as the information obtainable from multiple horse owners. Alternatively, one could attempt to differentiate cases and controls at screening age. Septic vascular failure is readily identifiable in histological sections, but this cannot be used for screening [25]. The most likely test to differentiate clinically would be polymerase chain reaction (PCR) on synovial fluid for a panel of common bacterial species [32]. This is something we would like to explore, because it could be useful in valuable Coldblooded trotter stallions [28]. It is unlikely to be feasible in large screening studies, and once a genetic test for osteochondrosis becomes available, clinical differentiation of septic and aseptic lesions is superfluous.

The sepsis cohort was compared to three different cohorts [17, 30, 31] selected because of representative populations and similar methods (Additional file 1: Table S1). A difference was found between the groups, and it is highly likely that the difference was due to bacterial infections [20–23, 25]. The study was subject to the same issues as other clinical, retrospective studies, and several confounding factors were considered. The infection status of the comparison cohorts was unknown [17, 30, 31]. Some lesions could therefore have been due to bacteria, for example in the horse with 6 lesions [17] (Table 4). As long as prevalence in the sepsis cohort was higher, this factor could have led to underestimation of the true difference between the groups, but it could not have compromised the conclusion. The sepsis cohort was older than the comparison cohorts at the time of screening. Lesions can resolve before certain joint-specific age thresholds [8, 9]. All horses were older than the upper age threshold for resolution of hock lesions in Standardbreds of 7/8 months [8]. The fact that the sepsis cohort was older therefore provided more time for lesions to resolve before screening [8, 9], underestimating the difference between the groups and therefore not compromising the conclusion. The prevalence of osteochondrosis may have increased, and radiological detection may have improved, between the different generations of foals. This would apply to the AMG [17] and JL [31] cohorts, but not the SL [30] comparison cohort. An increased plane of nutrition has been associated with increased lesion prevalence [33, 34]. The sepsis cohort suffered infections, some of which were associated with inappetence (Table 1). The sepsis cohort is therefore likely to have had the same or lower plane of nutrition than the comparison cohorts, leading to underestimation of the difference between the groups. Box-rested and exercised foals developed the same number of lesions, but lesions were more severe in box-rested than exercised foals [35]. Confinement during infections can therefore be a reason for more severe lesions in the sepsis cohort [35], but it should not influence the number of lesions and therefore not lead to over−/underestimation or an erroneous conclusion. On balance, this leads us to believe the current results may represent an underestimation of the true difference, but are likely to be internally valid and may also have external validity to Standardbred and other horse breed populations [36, 37].

Lesions in the sepsis cohort probably represent a mixture of septic, acquired [25] and aseptic, heritably predisposed disease [2]. The blood supply regresses from distal limb joints at an earlier age than proximal limb joints [1, 2, 38], and the growth cartilage of the fetlock is largely avascular at ~ 35 days in Standardbred foals [39]. Fetlock lesions in foals presenting with infections at ≥35 days therefore have to be the result of previous subclinical infections, or aseptic vascular failure. Although regression occurs earlier from distal than proximal limb joints, it is similar between left and right joints [1] and the most likely explanation for lesion symmetry is that the two joints in a pair have the same period of vulnerability to vascular failure. Finally, all predilection sites in the hock [2] and some predilection sites in the fetlock [39] are the last to lose their blood supply; they are therefore believed to be predilection sites because they have the longest periods of vulnerability. Septic and aseptic lesions appear to be confined to the same sites and behave similarly in terms of symmetry. This probably reflects the fact that the distribution of both categories of lesions is determined largely by the distribution of the blood supply at the time of the initiating vascular insult.

The distribution of the blood supply alone does not explain all aspects of the distribution of lesions. Aseptic vascular failure has been associated with the process of incorporating vessels into the advancing ossification front [2, 3]. Localisation may be further explained by variation in the quality of collagen type I fibres around individual vessels [40–42]. Incorporation happens a limited number of times and can therefore lead to a finite number of lesions [43, 44]. The trigger for septic vascular failure is probably a combination of bacterial binding affinity [45] and discontinuities in the vascular endothelium [22, 40, 46]. Discontinuities are constitutionally present and occur as part of normal development [47]. Bacterial toxins and inflammatory mediators also rapidly lead to opening of additional discontinuities [47]. Septic vascular failure can therefore potentially cause an infinite number of lesions. Bacteria were previously found bound to growth cartilage in other, distant joints 24 h after having been injected into the stifle of pigs [20]. Lesions were found in multiple joints in addition to the ones that had been diagnosed with septic arthritis in the current sepsis cohort (Table 2). The additional joints could have suffered subclinical sepsis, or mild sepsis masked by more marked sepsis in other affected joints, or they could be the result of opening of additional discontinuities in joints distant from the ones with clinical sepsis [20, 47]. The literature on sepsis-induced discontinuities prompted the extraction of new information from the SL cohort [30]. The sepsis cohort had more affected joints, and more lesions per affected horse than the SL cohort. Our interpretation is that this is because septic vascular failure is not confined to sites of vascular incorporation [2, 43, 44] but rather can occur anywhere endothelial discontinuities are present constitutionally or induced to open up [20, 40, 47]. We also believe this to be the most likely explanation for why some lesions subjectively appeared larger and more complex in the sepsis cohort (Fig. 2) than what is commonly reported in the literature [17, 30, 31]. It would have been interesting, but was not possible to compare potential differences in the relative prevalence of different fetlock lesions. If POFs genuinely are more common medially in heritably predisposed disease [17, 30, 31] and laterally in septic vascular failure, it is now possible to generate the hypothesis that this is because endothelial discontinuities are more prevalent laterally [22, 40, 46], and that biomechanical force has a more deleterious effect on collagen type I quality medially [40–42].

The number of permanent lesions is a result of the balance between lesions arising and resolving [8, 9, 48]. As discussed above, some of the current results (joints distant from the ones diagnosed with sepsis in 3/28 horses; complex lesions with > 1 fragment or bed in 3/28 horses) tentatively indicate that a greater number of lesions are initiated in septic versus aseptic vascular failure. Some heritably predisposed lesions appear inert and stimulate little or no secondary response [49, 50]. Septic vascular failure stimulates more of an inflammatory response, including extravasation of neutrophils within cartilage canals [25]. If the increased inflammatory response leads to more successful resolution, the number of permanent lesions could be reduced compared to aseptic vascular failure. It seems unlikely that resolution is increased in septic versus aseptic vascular failure, as the number of permanent lesions was indeed increased. The relative contributions of more lesions arising and fewer lesions resolving to increased prevalence of septic lesions may seem academic, but could prove clinically relevant in terms of diagnosis, staging and treatment planning. The proportions would, however, have to be determined using a different study design.

## Conclusions

Standardbred horses that survived bacterial infections before 6 months of age had more osteochondral lesions than literature comparison cohorts at screening age. The implication was that some of the lesions in this group were caused by bacteria. It may become necessary to develop methods for differentiating between acquired, septic and aseptic, heritably predisposed lesions.

## Additional file

Additional file 1: **Table S1.** Literature search. (DOCX 27 kb)

## Abbreviations

AMG: Ann Margaret Grøndahl
CI: Confidence interval
D: Dorso-proximal
DP1: Dorso-proximal first phalanx fragments
EH: Eli Helene Støkken Hendrickson
JP: Jan Philipsson
NID: Nils Ivar Dolvik
OCD: Osteochondrosis dissecans
P1: First phalanx
PCR: Polymerase chain reaction
POF: Palmaro−/plantaro-proximal osteochondral fragment
SL: Sigrid Lykkjen
UPE: Ununited palmaro−/plantaro-proximal eminence
