# Supplementary material for: Prevalence of osteochondral lesions in the fetlock and hock joints of Standardbred horses that survived bacterial infection before 6 months of age
Source: BMC Vet Res. 2018 Dec 10;14:390. doi: 10.1186/s12917-018-1726-3 (PMC6288956; doi:10.1186/s12917-018-1726-3)
Supplement: Supplementary file 1 — Table S1. Literature search. (DOCX 27 kb) [file 12917_2018_1726_MOESM1_ESM.docx]

**Additional file 1: Table S1. Literature search**

| **Citation** | **Standardbreds examined** | **Population source** | **Screening age** | **Radiographic projections** | **Lesions registered** | **Prevalence** | **Included or excluded** |
| --- | --- | --- | --- | --- | --- | --- | --- |
| *Historical, earliest references:* | | | | | | | |
| Falk-Rønne & Kristoffersen, 1980 [1] | 133 Danish Standardbreds | 1 training yard | 2 year-olds and 3 year-olds | Hock only: DP^a^ and D45°MPLO^b^ | DIRT^c^, LTR^d^, MM^e^ | 18/133 horses (13.5 %) | Excluded due to horse number |
| Hoppe, 1984 [2] | 208 Gotland + 130 Uppsala Swedish Standardbreds | Random sampling | Foal age to 4 years old | Hocks only: D45°MPLO | DIRT, LTR, lateral malleolus | 55/338 Standardbred horses (16.3 %) | Excluded due to horse number |
| *Heritability studies:* | | | | | | | |
| Grøndahl & Dolvik, 1993 [3] | 753 Norwegian Standardbreds | 60 % of 1988 foal crop, 644 were progeny from 39 sires with > 5 offspring | 6-21 months; 86 % between 8-13 months | 8 total: D45°MPLO hocks, LM^f^ front fetlocks, D35°Pr45°L/MO^g^ hind fetlocks | DIRT, LTR, SR^h^, DP1^i^, POF^j^ (UPE^k^ reported separately) | 318/753 horses (42.2 %) | Included as AMG cohort |
| Philipsson *et al.*, 1993 [4] | 793 Swedish Standardbreds | Progeny groups from stallions with > 20 offspring | 11-23.7 months, average: 17 months | 8 total: D45°MPLO hocks, LM front fetlocks, D35°Pr45°L/MO hind fetlocks | DIRT, LTR, SR + DP1 reported together, POF, UPE | 285/793 horses (35.9 %) | Included as JP cohort |
| Lykkjen *et al.*, 2012 [5] | 363 Norwegian Standardbreds | Progeny groups from stallions with > 10 offspring | 9-18 months, mean: 12.4 ± 1.9 months | 10 total: D45°MPLO, D30°LPMO^l^ hocks, LM front fetlocks, D35°Pr45°L/MO hind fetlocks | DIRT, LTR, MM, SR, DP1, POF, UPE | 179/363 horses (50.7 %) | Included as SL cohort |
| *Performance studies:* | | | | | | | |
| Brehm & Staecker, 1999 [6] | 1190 German Standardbreds | Hospital population | Not given | Hocks only: LM, D45°LPMO, D45°MPLO | DIRT, LTR | 147/1190 horses (12.4 %) | Excluded due to hocks only |
| Torre & Motta, 2000 [7] | 764 Italian Standardbreds | Yearling sales | Yearlings | 6 views total: D45°LPMO, D45°MPLO hocks, LM all four fetlocks | DIRT, LTR, MM, SR, DP1, POF, UPE, PSB^m^ | 186/764 horses (24 %) | Excluded due to different projections |
| Couroucé-Malblanc *et al.*, 2006 [8] | 865 French Standardbreds | 20 training yards | 23.5 ± 2.2 months | 6 views total: LM all four fetlocks, LM hock, LM stifle | 12 abnormalities: DSB^n^, P3^o^, DIPJ^p^, PIPJ^q^, foot, front fetlock, PIPJ, dorsal/plantar fetlock, proximal/distal hock, stifle | 363/865 horses (41.9 %) | Excluded due to different projections and lesion scoring |
| Langlois *et al.*, 2006 [9] | 740 French Standardbreds | Yearling sales/before training | 16-18 months old | 6 views total: LM of all four fetlocks and both hocks | Osteochondrosis, OCD^r^, cysts, epiphysitis | 76/740 horses (10.3 %) | Excluded due to different projections and lesion scoring |
| Lepeule *et al.*, 2008 [10], 2009 [11] and The Veterinary Journal, 2013 [12] | 161 French Standardbreds | BOSAC study | 172 days ± 38 days | 10 views total: DP carpi, LM all four digits, LM tarsi, LM stifles | Large range of juvenile osteochondral conditions evaluated | Not possible to extract just Standardbred OCD prevalence | Excluded due to different projections and lesion scoring |

^a^DP: Dorso-plantar. ^b^D45°MPLO: Dorsal-45°-medial plantaro-lateral oblique. ^c^DIRT: Cranial distal intermediate ridge of tibia. ^d^LTR: Lateral trochlear ridge of talus. ^e^MM: Medial malleolus of tibia. ^f^LM: Latero-medial. ^g^D35°Pr45°L/MO: Dorsal-35°-proximal-45°-lateral/medial to plantaro-distal-medial/lateral oblique. ^h^SR: Sagittal ridge of third metacarpal/metatarsal bone. ^i^DP1: Dorso-proximal first phalanx. ^j^POF: Palmaro-/plantaro-proximal osteochondral fragment. ^k^UPE: Ununited palmaro-/plantaro-proximal eminence. ^l^D30°LPMO: Dorsal-30°-lateral plantaro-medial oblique. ^m^PSB: Proximal sesamoid bones. ^n^DSB: Distal sesamoid bone. ^o^P3: Third phalanx. ^p^DIPJ: Distal interphalangeal joint. ^q^PIPJ: Proximal interphalangeal joint. ^r^OCD: Osteochondrosis dissecans.

**References**

1. Falk-Rønne J, Kristensen AR: **Forekomsten af osteochondrose i talo-cruralleddet hos unge travheste i træning (Prevalence of osteochondrosis in the talo-crural joint of young trotters in training)**. *Dansk Vet Tidsskr* 1980, **63**(4):141-143.

2. Hoppe F: **Radiological investigations of osteochondrosis dissecans in Standardbred Trotters and Swedish Warmblood horses**. *Equine Vet J* 1984, **16**(5):425-429.

3. Grøndahl AM, Dolvik NI: **Heritability estimations of osteochondrosis in the tibiotarsal joint and of bony fragments in the palmar/plantar portion of the metacarpo- and metatarsophalangeal joints of horses**. *J Am Vet Med Assoc* 1993, **203**(1):101-104.

4. Philipsson J, Andréasson E, Sandgren B, Dalin G, Carlsten J: **Osteochondrosis in the tarsocrural joint and osteochondral fragments in the fetlock joints in Standardbred trotters. II. Heritability**. *Equine Vet J Suppl 16* 1993:38-41.

5. Lykkjen S, Roed KH, Dolvik NI: **Osteochondrosis and osteochondral fragments in Standardbred trotters: Prevalence and relationships**. *Equine Vet J* 2012, **44**(3):332-338.

6. Brehm W, Staecker W: **Osteochondrosis (OCD) in the tarsocrural joint of Standardbred trotters - correlation between radiographic findings and racing performance**. In: *American Association of Equine Practitioners: 1999; Albuquerque, New Mexico*; 1999: 164-166.

7. Torre F, Motta M: **Osteochondrosis of the tarsocrural joint and osteochondral fragments in the fetlock joints: incidence and influence on racing performance in a selected group of Standardbred trotters**. In: *American Association of Equine Practitioners: 2000; San Antonio, USA*; 2000: 287-294.

8. Couroucé-Malblanc A, Leleu C, Bouchilloux M, Geffroy O: **Abnormal radiographic findings in 865 French Standardbred trotters and their relationship to racing performance**. *Equine Vet J Suppl 36* 2006:417-422.

9. Langlois B, Blouin C, Perrocheau M, Chaffaux S: **Influence of radiographic osteochondrosis status at 16-18 months, on racing performances in French trotters**. *Pferdeheilk* 2006, **22**(4):461-464.

10. Lepeule J, Bareille N, Valette JP, Seegers H, Jacquet S, Denoix JM, Robert C: **Developmental orthopaedic disease in limbs of foals: between-breed variations in the prevalence, location and severity at weaning**. *Animal* 2008, **2**(2):284-291.

11. Lepeule J, Bareille N, Robert C, Ezanno P, Valette JP, Jacquet S, Blanchard G, Denoix JM, Seegers H: **Association of growth, feeding practices and exercise conditions with the prevalence of Developmental Orthopaedic Disease in limbs of French foals at weaning**. *Preventive veterinary medicine* 2009, **89**(3-4):167-177.

12. Jeffcott LB, Mills PE: **Special Issue: Equine Juvenile Osteochondral Conditions**. *The Veterinary Journal* 2013, **197**(1):1-111.
